# Liquid- and Air-Filled Catheters without Balloon as an Alternative to the Air-Filled Balloon Catheter for Measurement of Esophageal Pressure

**DOI:** 10.1371/journal.pone.0103057

**Published:** 2014-09-23

**Authors:** Alessandro Beda, Andreas Güldner, Alysson R. Carvalho, Walter Araujo Zin, Nadja C. Carvalho, Robert Huhle, Antonio Giannella-Neto, Thea Koch, Marcelo Gama de Abreu

**Affiliations:** 1 Pulmonary Engineering Group, Clinic of Anaesthesiology and Intensive Care Therapy, University Hospital Dresden, Technische Universität Dresden, Dresden, Germany; 2 Department of Electronic Engineering and Postgraduate Program of Electrical Engineering, Federal University of Minas Gerais, Pampulha, Belo Horizonte, Brazil; 3 Laboratory of Respiration Physiology, Carlos Chagas Filho Biophysics Institute, Universidade Federal do Rio de Janeiro, Rio de Janeiro, Brazil; 4 Biomedical Engineering Program, Alberto Luis Coimbra Program of Post-Graduation and Research in Engineering, Universidade Federal do Rio de Janeiro, Rio de Janeiro, Brazil; Vanderbilt University Medical Center, United States of America

## Abstract

**Background:**

Measuring esophageal pressure (P_es_) using an air-filled balloon catheter (BC) is the common approach to estimate pleural pressure and related parameters. However, P_es_ is not routinely measured in mechanically ventilated patients, partly due to technical and practical limitations and difficulties. This study aimed at comparing the conventional BC with two alternative methods for P_es_ measurement, liquid-filled and air-filled catheters without balloon (LFC and AFC), during mechanical ventilation with and without spontaneous breathing activity. Seven female juvenile pigs (32–42 kg) were anesthetized, orotracheally intubated, and a bundle of an AFC, LFC, and BC was inserted in the esophagus. Controlled and assisted mechanical ventilation were applied with positive end-expiratory pressures of 5 and 15 cmH_2_O, and driving pressures of 10 and 20 cmH_2_O, in supine and lateral decubitus.

**Main Results:**

Cardiogenic noise in BC tracings was much larger (up to 25% of total power of P_es_ signal) than in AFC and LFC (<3%). Lung and chest wall elastance, pressure-time product, inspiratory work of breathing, inspiratory change and end-expiratory value of transpulmonary pressure were estimated. The three catheters allowed detecting similar changes in these parameters between different ventilation settings. However, a non-negligible and significant bias between estimates from BC and those from AFC and LFC was observed in several instances.

**Conclusions:**

In anesthetized and mechanically ventilated pigs, the three catheters are equivalent when the aim is to detect changes in P_es_ and related parameters between different conditions, but possibly not when the absolute value of the estimated parameters is of paramount importance. Due to a better signal-to-noise ratio, and considering its practical advantages in terms of easier calibration and simpler acquisition setup, LFC may prove interesting for clinical use.

## Introduction

The assessment of several mechanical and functional properties of the lungs and chest wall depends on the estimation of pleural pressure (P_pl_). The end-expiratory transpulmonary pressure (P_trans_, the difference between alveolar pressure and P_pl_) has been used for positive end-expiratory pressure (PEEP) adjustment in mechanically ventilated patients [1]. Estimates of P_pl_ are also necessary to evaluate respiratory effort and work of breathing, for example during assisted mechanical ventilation (MV) and weaning from MV, in order to detect the occurrence of respiratory fatigue [2]–[4]. Furthermore, P_pl_ can be used to split estimates of total respiratory system elastance into its pulmonary and chest wall components [2].

In clinical practice, P_pl_ is estimated from the esophageal pressure (P_es_). Usually, P_es_ is measured with an air-filled balloon catheter inserted in the middle portion of the esophagus [5], [6]. Several investigations reported a good agreement between measurement of the changes in P_pl_ and P_es_ [7]–[9]. Noteworthily, other studies reported that an offset exists between absolute values of P_pl_ and P_es_, and that the agreement between the changes in those two pressures is reduced in mechanically ventilated subjects, supine position, and injured (i.e. more heterogeneous) lungs [10]–[14].

Despite its potential benefits, P_es_ is not routinely measured in mechanically ventilated patients [4]. This is partly related to the following factors: 1) complex calibration, involving balloon filling and maneuvers on the patient; 2) contamination of the measurements by cardiogenic and movement artifacts; and 3) need of repositioning and recalibration due to signal quality loss over time [4], [8], [15], [16]. Thus, alternative technologies that improve the signal-to-noise ratio of P_es_ measurement and simplify its use at the bedside are desirable.

The main aim of this study was to compare the traditional air-filled balloon catheter technique with liquid-filled and air-filled catheters without balloon for measurement of P_es_ during controlled MV, as well as MV with spontaneous breathing, in juvenile pigs without lung injury. We calculated the partitioned elastance (E_L_ for lung and E_cw_ for chest wall), transpulmonary pressure (P_L_), esophageal pressure time product (PTP), and the inspiratory work of breathing (WOBi) with each of those catheter techniques and assessed the differences. We hypothesized that both the liquid-filled and air-filled catheters without balloon can be used interchangeably with the traditional air-filled balloon catheter technique for monitoring of P_es_, and estimating related parameters.

## Materials and Methods

### Experimental protocol

After ethical approval by the local authorities (Landesdirektion Sachsen, Dresden, Germany, reg. nr. 24-9168.11-1/2009-27), seven healthy female juvenile pigs (32–42 kg) were intravenously anesthetized (propofol 2–7 mg/kg/h, sufentanil 0.3–1.5 µg/kg/h) and tracheally intubated with a cuffed tube (8.0 mm inner diameter).

After intubation, the animals were mechanically ventilated using an *EVITA XL* ventilator (*Dräger*, Germany) with biphasic positive airway pressure/airway pressure release ventilation (BIPAP/APRV) mode (FiO_2_ = 1), which consists in transitions between two levels of airways pressure (P_low_ and P_high_) at a fixed rate. After the administration of a bolus of atracurium bromide (1 mg/kg) to abolish spontaneous breaths, the animals underwent a sequence of changes in the levels of PEEP ( = P_low_) and driving pressure (ΔP = P_high_–P_low_), resulting in the following combinations of PEEP/ΔP: 5/10, 5/20, 15/10, and 15/20 cmH_2_O. Inspiration and expiration durations were fixed to last 1 s in all conditions. All steps of the sequence lasted 2 minutes and were performed first in supine and then in right lateral decubitus. The absence of spontaneous breathing in this phase was monitored by visual inspection of P_es_ tracing. When muscle relaxation ceased and consistent inspiratory efforts appeared in the P_es_ tracings, the animals were kept in right lateral decubitus under BIPAP/APRV ventilation (PEEP = 5 cmH_2_O, ΔP = 10 cmH_2_O), but allowing unsupported spontaneous breaths, with inspiratory time of 1 s and expiratory time adjusted manually to permit three or more spontaneous breaths after each controlled cycle. This step lasted approximately 4 minutes and was then repeated in the supine decubitus. The animals were then euthanized with an i.v. injection of thiopental (2 g) and KCl 1 M (50 mL).

**Figure 1:**
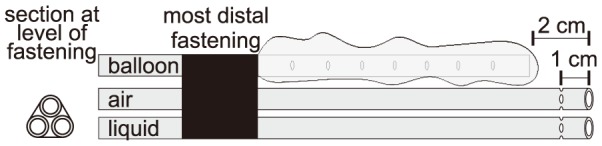
Illustration of the combination of catheters used for the experiment. The catheters were permanently fastened together, with the tip of the air- and liquid-filled catheters positioned 2 cm further down the esophagus than the tip of the balloon. Additional 4 holes were created in the liquid- and air-filled catheters to minimize the possibility of obstruction, located equidistantly along the circumference of the catheter at approximately 1 cm from the tip.

Three catheters were adopted for esophageal pressure measurement: a conventional commercially available balloon catheter (*Cardinal Health*, USA); an air-filled catheter, obtained by removing the balloon portion from the previous catheter; a liquid-filled catheter, adapted from the disposable tubing commonly used for invasive blood pressure measurement (taken from the *DTXPlus* kit, *Becton Dickinson*, USA) by removing its distal Luer-lock adapter and creating four holes near the tip, as illustrated in Figure 1. A constant flow of saline solution of approximately 3 ml/h was maintained in the catheter. The three catheters were bundled in a combined catheter, as illustrated in Figure 1, and introduced in the esophagus. The inner diameter was 2.0 mm for the liquid-filled catheter and 2.6 mm for the others. Due to their mechanical properties, all catheters can be considered as rigid tubes for what concerns the propagation of pressure waves in the range of measured P_es_. Bench tests were performed by placing the bundle of catheters horizontally at the bottom of a recipient and increasing the water level from 0 to 5, 15, and 20 cm. Pressure measurement errors were always <5%, and the differences between catheters were always <1%.

### Data acquisition and processing

The correct positioning of the catheters in the esophagus was achieved using the signal of the air-filled balloon catheter as a reference, according to the procedure previously described [5]. Briefly, after inserting the empty balloon catheter in the stomach and injecting 0.5 mL of air into the balloon, the catheter was withdrawn slowly until P_es_ oscillations appeared and were in phase with airways pressure oscillations (i.e. balloon in the esophagus). Following that, the catheter was drawn a further 10 cm, in order to position the balloon midway between the apex and the base of the lungs. Occlusion and chest compression maneuvers were then used to correct the final position of the balloon and the amount of air in the balloon catheter in order to obtain a ratio between P_es_ and airways opening pressure of ∼1.

These maneuvers were performed at the beginning of the experiment during controlled MV in the paralyzed animals. Occlusion maneuvers during spontaneous breathing efforts, as previously described [7], were performed before and after the assisted ventilation task, which confirmed in all cases that repositioning and recalibration of the catheters were not necessary. The balloon catheter, air-filled catheter, and airway opening were connected to gas pressure transducers (*163PC01D48-PCB*, *Sensortechnics GmbH*, Germany). The liquid-filled catheter was connected to a conventional disposable blood pressure transducer (*DTXPlus*, *Becton Dickinson*, USA), positioned at the height of the midaxillary line (expected height of the tip of the catheter in the esophagus). The outputs of all transducers were linearly related to pressure in the range of interest, and were calibrated before the experiments adopting a linear regression approach, using several pressure levels obtained with gas pressure generators or water columns. These signal, together with one derivation of EKG obtained with a biosignal amplifier (LP511, *Grass Technologies*, USA), were synchronously acquired using a data acquisition card (*NI USB-6210*, *National Instruments*, USA) with a sampling frequency of 1000 Hz.

Airflow and airway opening pressure signals were continuously acquired from the ventilator and fed into the PC through a serial interface (sampling frequency of 125 Hz), and synchronized off-line with the other signals (by means of automatic time alignment, using maximal covariance, between the airway pressure signal acquired using the data acquisition system and the airway pressure signal that could also be retrieved from the ventilator), after resampling all signals at 100 Hz.

The onset of inspiration and expiration were automatically detected in the airflow signal, and then manually edited. The cycles were manually labeled as controlled or spontaneous, and for the latter the onset of the inspiratory effort was identified in the esophageal pressure tracings (onset of a drop larger than 2 cmH_2_O occurring right before the onset of inspiration). P_trans_ was computed as the difference between the airways opening pressure and P_es_ measured with each catheter.

**Figure 2:**
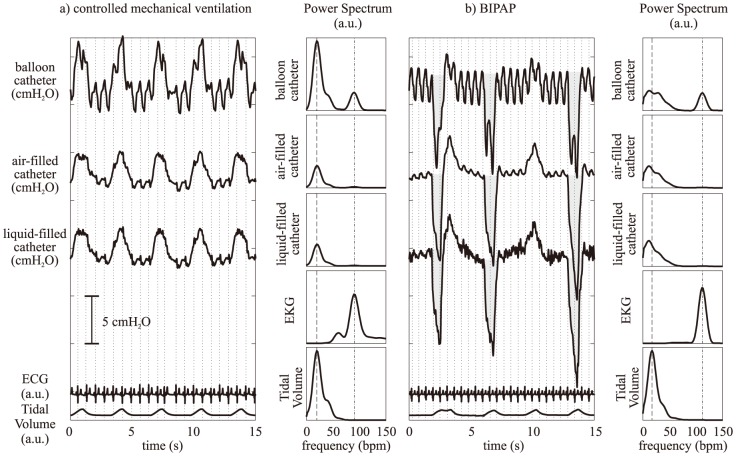
Representative esophageal pressure measurement tracings during a) controlled mechanical ventilation and b) biphasic positive airway pressure (BIPAP) ventilation with spontaneous breathing (note: offsets between the tracings are just for pictorial representation). The balloon catheter tracings show large oscillations that are coherent with the heart-beats occurrences in the EKG (the dotted vertical lines represent the occurrence of the R-peaks of the EKG, i.e. ventricular contraction). To the right side of the tracings the power spectra of each esophageal pressure signal is shown, disclosing that the largest amount of the power is concentrated at frequencies compatible with the respiratory spectrum (dashed line represents the average respiratory rate). Interestingly, only the balloon catheter presents a peak of considerable power at frequencies compatible with the EKG spectrum (the dash-dot lines represent the average heart rate). The pressure-time product (PTP) is graphically represented by the gray areas in panel b): in the balloon tracings it is evident that cardiogenic noise affects the shape of the area, modifying the estimated value of PTP.

In each respiratory cycle, several indexes were estimated using the P_es_ signal obtained from each catheter. From the P_trans_ tracings, the end-expiratory value and the inspiratory change were computed. The dynamic lung and chest wall elastance (E_L_ and E_cw_, respectively) were estimated from pressure, volume, and flow signals using a least-squares identification of the coefficients of the conventional linear unicompartmental models of lung mechanics shown in Eqs. 1 and 2:



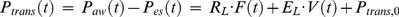
(Eq.1)




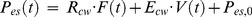
(Eq.2)where R_L_ and R_cw_ represent resistance to airflow, F is airflow, V is volume, and P_es,0_ and P_trans,0_ are the values of P_es_ and P_trans_ when F = 0 and V = 0, respectively. E_cw_ estimates were not considered for the spontaneous breaths, since E_cw_ possess a physiological meaning only when the chest wall is a ‘passive’ structure, as in controlled ventilation, which is not the case for spontaneous breaths (resulting in misleading lower E_cw_ estimates). For the spontaneous breaths only, the pressure time product (PTP) was estimated for each cycle, as shown in Eq. 3:



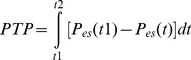
(Eq.3)where t1 corresponds to the onset of the inspiratory effort, and t2 corresponds to the time when P_es_ returns to its value at t1 (as illustrated in Figure 2-b). The inspiratory work of breathing (WOBi) was estimated for each inspiration based on the Campbell diagram [2], [3]. Average values of the parameters were computed for each subject in the following conditions: controlled breaths during BIPAP/APRV without spontaneous breathing (average of 60 breaths); controlled breaths during BIPAP/APRV with spontaneous breathing (average of 20 breaths); spontaneous unsupported breaths during BIPAP/APRV with spontaneous breathing (average of 80 breaths).

### Statistical analysis

During BIPAP/APRV without spontaneous breathing, a general linear model with Greenhouse-Geisser correction was used to test the effect on the average value of each estimated parameter of the method to measure P_es_, considering position (lateral/supine), PEEP (5/15 cmH_2_O), and ΔP (10/20 cmH_2_O). During BIPAP/APRV with spontaneous breaths, the same analysis was repeated separately for controlled and spontaneous cycles (and disregarding the PEEP and ΔP factors, which were fixed). The amount of cardiogenic noise in each P_es_ tracing was quantified as the percentage of power of the signal in the frequency band [HR-10 HR+10], where HR is the average heart rate (in bpm) computed from the EKG (power spectrum was computed using Welch’s modified periodogram method [17], using multiple segments corresponding to 40 s of data, a 90% overlap between adjacent segments, and a Hanning windowing). The agreement between estimates of the parameters obtained using different catheters was assessed through the graphical method introduced by Bland and Altman [18], and quantified by means of bias and precision (mean and SD of difference between two methods, respectively). Additionally, the difference between the parameters estimated using different methods to measure P_es_ was tested using paired t-tests. All tests were performed with the software SPSS (IBM, USA), and statistical significance was accepted at p<0.05.

## Results

Figure 2 shows a representative example of P_es_ tracings obtained with the three catheters, during BIPAP/APRV with and without spontaneous breaths. While the inspiratory/expiratory changes are roughly similar in all instances, the measurement obtained with the air-filled balloon catheter is more contaminated by cardiogenic noise than the other methods. Such phenomenon is also highlighted by the spectra of EKG and balloon signals, showing a coincident large peak at the heart frequency. This was a consistent feature in the tracings of all subjects. In fact, while for the catheters without balloon the fraction of P_es_ power related to cardiogenic oscillations was only <3%, for the balloon catheter it was>25% during controlled ventilation, and>15% during BIPAP.

**Figure 3:**
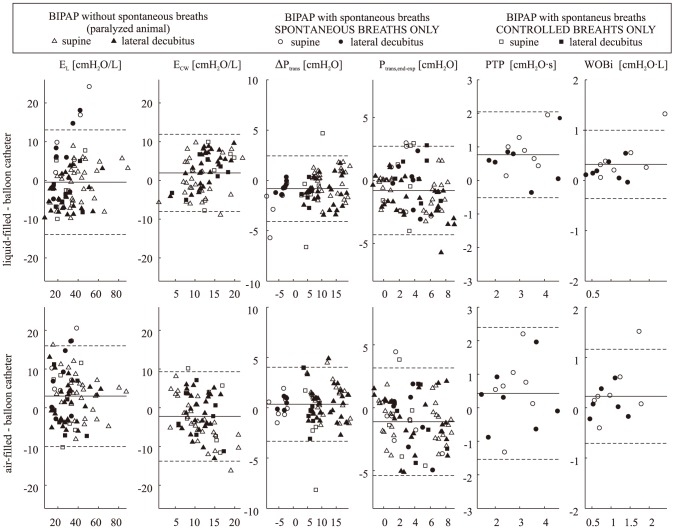
Bland-Altman plots comparing average parameters estimated using the balloon catheter measurement with those obtained using the air- or liquid-filled catheter, under different settings of BIPAP (biphasic positive airway pressure) mechanical ventilation. E_L_, E_cw_: lung and chest wall elastance; ΔP_trans_: inspiratory change in transpulmonary pressure; P_trans,end-exp_: end-expiratory value of transpulmonary pressure; PTP: pressure-time product; WOB_i_: inspiratory work of breathing. The horizontal lines represent the mean (solid line) ± SD (dashed lines) of the difference between methods.

Figure 3 shows Bland-Altman plots comparing the estimate of each parameter obtained using the balloon measurements with those obtained using the other two catheters. A considerable dispersion can be noted in all instances, which does not seem to be affected by the magnitude of the estimated parameter. Significant bias was detected in several cases, as reported in Table 1.

**Table 1:** Bias (precision) of parameters estimated using the liquid- or air-filled catheters compared to those obtained with the balloon catheter, in different conditions.

balloon Vs.	E_L_			E_cw_			ΔP_trans_			P_trans,endexp_			PTP			WOB_i_	
	[cmH_2_O/L]			[cmH_2_O/L]			[cmH_2_O]			[cmH_2_O]			[cmH_2_O•s]			[cmH_2_O•L]	
spont BIPAP	7.9	(8.2)	**				−1.3	(1.5)	**	−0.3	(2.2)		0.8	(0.6)	***	0.3	(0.3)
contr BIPAP	−2.7	(5.3)		1.8	(5.3)		−1.0	(2.4)		0.1	(2.2)						
contr MV	−2.0	(5.0)	**	2.0	(5.0)	**	−0.6	(1.4)	**	−1.1	(1.4)	***					
spont BIPAP	7.7	(7.6)	**				0.3	(0.9)		−0.9	(2.4)		0.4	(1.0)		0.2	(0.5)
contr BIPAP	0.8	(7.4)		−1.6	(7.0)		−0.5	(3.0)		−0.9	(2.5)						
contr MV	2.4	(5.5)	**	−2.4	(5.5)	**	0.6	(1.6)	*	−1.2	(2.0)	***					

BIPAP: biphasic positive airway pressure; contr MV: controlled mechanical ventilation (with animal paralyzed using atracurium bromide); contr BIPAP: assisted ventilation (allowing unsupported spontaneous breaths), considering only the controlled breaths; spont BIPAP: assisted ventilation, considering only the unsupported spontaneous breaths. E_L_, E_cw_: lung and chest wall elastance; ΔP_trans_: inspiratory change in transpulmonary pressure; P_trans,end-exp_: end-expiratory value of transpulmonary pressure; PTP: pressure-time product; WOB_i_: inspiratory work of breathing. Significance of t-test:

*p<0.05;

**p<0.01;

***p<0.001.

**Figure 4:**
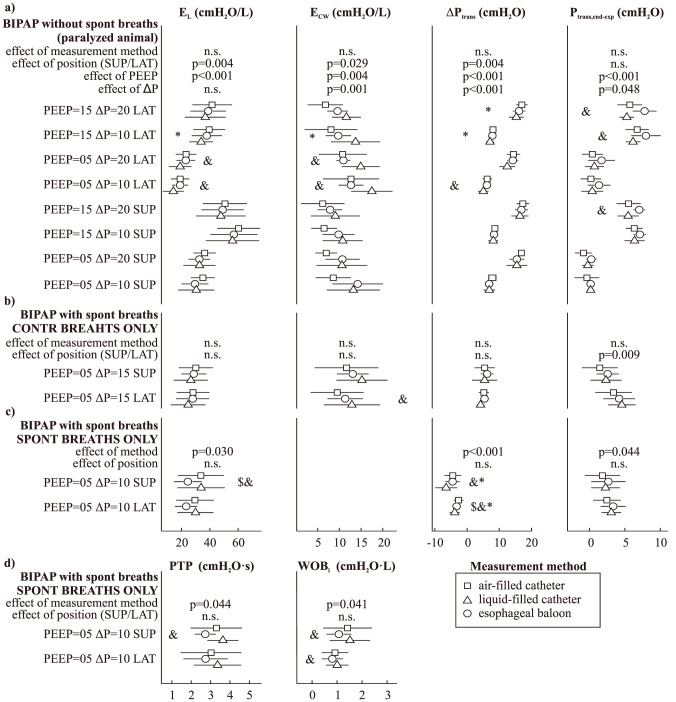
Average value (±SD) of the parameters derived from esophageal pressure measurements under different settings of BIPAP (biphasic positive airway pressure) mechanical ventilation: a) controlled ventilation (with animal paralyzed using atracurium bromide); b) assisted ventilation (allowing unsupported spontaneous breaths), considering only the controlled breaths; c) and d) assisted ventilation, considering only the unsupported spontaneous breaths. PEEP: positive end-expiratory pressure (cmH_2_O); ΔP: driving pressure (cmH_2_O); SUP, LAT: supine or right lateral decubitus; E_L_, E_cw_: lung and chest wall elastance; ΔP_trans_: inspiratory change in transpulmonary pressure; P_trans,end-exp_: end-expiratory value of transpulmonary pressure; PTP: pressure-time product; WOB_i_: inspiratory work of breathing. The p-value of the effect of measurement method, position (SUP/LAT), PEEP and ΔP was computed using a general linear model approach (n.s. corresponds to p-value>0.05). &: significant (p<0.05) difference between indexes estimated using the air-filled and liquid-filled catheter, using a paired t-test. Similar notation for the comparisons of the balloon catheter with the air-filled ($) and liquid-filled (*) catheter.

Figure 4 illustrates the average estimate of each parameter using the three different measurements of P_es_, for each combination of ventilation settings and position tested. The effect of the method to measure P_es_ was not significant when only controlled breaths were considered (independently of spontaneous breaths being allowed or not). Conversely, a significant effect was found when solely the unsupported spontaneous breaths were employed. In particular, the balloon catheter measurement produced somewhat lower E_L_, PTP and WOBi estimates during spontaneous breaths. Also, for BIPAP without spontaneous breathing a significant influence of PEEP, ΔP, and position was found for all parameters (except for a non-significant effect of ΔP on E_L_). For BIPAP allowing unsupported spontaneous breaths a consistent position-dependent effect was not detected.

## Discussion

The main findings of this study were that in anesthetized and mechanically ventilated pigs: 1) P_es_ measured with the balloon catheter showed more contamination by cardiogenic noise than the methods without balloon; 2) in particular, such a noise resulted in consistently lower estimates of E_L_, PTP and WOBi during spontaneous breathing when using the balloon catheter, as compared to the other catheters; 3) differences between P_es_ derived parameters showed considerable dispersion, independently of the catheter considered; 4) despite dispersion and bias, all three methods allowed to detect similar changes in E_L_, PTP, WOBi, inspiratory change and end-expiratory value of transpulmonary pressure between different ventilation settings and positions.

Our primary objective was to assess the viability of techniques alternative to the balloon for the measurement of esophageal pressure in a clinical routine scenario. Two alternatives were considered: liquid-filled and air-filled catheters, which have been previously proposed [6], [9], [19]–[22], but have not found a widespread clinical acceptance, possibly due to the lack of a comprehensive validation study. To compare the techniques, we considered not only the differences in the recorded tracings, but also in several clinically relevant derived indexes, and in the practical difficulties in positioning, calibrating, and managing that can discourage their adoption in the clinical practice.

Overall, the agreement of parameters estimated using the balloon catheter with those measured with air- or liquid-filled catheters is far from ideal. A considerable dispersion was found in all instances, associated in some cases with non-negligible bias. This indicates that the parameters estimated with the air-filled and liquid-filled catheter might not be interchangeable with the balloon catheters when comparing the absolute value of the parameters estimated. However, it is debatable which of the three methods, if any, should be considered the “gold standard”. One notable finding should be mentioned. Even if the phenomenon does not reach statistical significance in all cases, absolute end-expiratory transpulmonary pressure estimated using the balloon catheter appears to be somehow larger in several cases. This is likely related to the fact that the balloon technique tends to measure the lowest pressure found among the several holes present in the catheter along the length of the balloon [23], resulting in a possibly small but negative offset in P_es_ estimation compared to the other two methods, in which the holes are only near or at the tip the catheter. Adding side holes along the length of the other two catheters should lead to a similar behavior of absolute P_es_ measurement, if such behavior is desired. However, the results of several investigation question the use of P_es_ for estimating absolute values of P_pl_, since a bias exists between the two at the same location, which can vary with posture and catheter positioning [11], [12], [24]. Also P_pl_ changes considerably between different locations due to gravitational effects and inhomogeneity of the lungs [4], [25]. Such topology of the absolute value of P_pl_ cannot be estimated by P_es_. In this context, the limitations regarding the possibility of estimating P_pl_ absolute values are intrinsic to the use of P_es_ and hence common to all the possible methods/catheters to measure it. Hence, as summarized by Drummond et al., "great attention to absolute pressure measurements in the esophagus is not justified", while the changes in P_pl_ can be estimated satisfactorily using P_es_, at least for the case of a healthy homogenous lung [24]. However, the offset in absolute measurement among methods might be relevant for other applications, such as the PEEP tuning strategy introduced by Talmor et al. [1], which is based on the simplifying assumption that P_pl_ is the same for the whole lung and is 5 cmH_2_O smaller than P_es_ in all conditions.

One source of the dispersion among estimates was the cardiogenic artifacts, which in our tests were generally much larger in the balloon tracings, and represent the most persistent and notable difference among the methods considered.

We speculate that this finding is related to the fact that the balloon is a closed and deformable system. Each ventricular contraction produces the following sequence of events: 1) transient reduction in the volume of the heart; 2) mechanical propagation of part of such deformation to the esophagus; 3) very small increase in the esophageal lumen, with a negligible decrease in P_es_; 4) very small transient increase in the internal volume of the balloon, which adheres to part of the internal surface of the esophagus (possibly "glued" to it by a layer of secretion) and consequently follows somewhat its deformation; 5) transient decrease of the pressure within the balloon (since the system is closed) but not in P_es_. In this context, the balloon acts partly as a transducer/amplifier of the changes in the geometry of the esophagus unrelated to esophageal pressure, rather than simply transmitting P_es_ oscillations. Considering that the balloon catheter is a closed system, changes in its internal pressure and volume (ΔP and ΔV) from a given state (P_0_,V_0_) must obey the equation (P_0_+ΔP)•(V_0_+ΔV) = P_0_•V_0_, and consequently ΔP = P_0_•ΔV/•(V_0_+ΔV). If a small change in volume is considered (ΔV<<V_0_), then ΔP≈-P_0_•ΔV/V_0_. Considering the reasonable simplification that P_0_ is approximately the atmospheric pressure ( = 1031 cmH_2_O), the previous equation shows that changes in the internal volume of the balloon as small as 0.1% (i.e. ΔV/V = 0.001) are capable of generating swings of about 1cmH_2_0. Such swings are compatible with our results, and are unrelated to those in the pressure outside the balloon (P_es_, in our case), since they reflect solely the mechanical deformations of the internal volume of the balloon, originated by cardiogenic mechanical excitation. Conversely, being the air- and liquid-filled catheters undeformable and open systems, this effect cannot be observed.

The cardiogenic artifacts are likely to result in larger estimation errors of the parameters using the balloon catheter, which is considerably more affected by this problem according to our results. One example is the estimation of PTP and WOBi. This estimation is based on the area below/above the P_es_ curve (expressed as function of time or volume, respectively), which is distorted by cardiogenic noise (as exemplified in Figure 2). Even if complex algorithms can be applied in an attempt to reduce (but not cancel) cardiogenic oscillations [26], [27], adopting air- or liquid-filled catheters seems a more practical option in this context. However, we cannot exclude that the discrepancy was exacerbated by the fact that in piglets the heart is relatively larger and nearer to the esophagus than in adult humans.

In spite of that, all the three measuring techniques allowed detecting changes of the same magnitude and direction when MV settings are modified. The results are compatible with the existing literature regarding the effect of PEEP and V_T_ on lung and chest wall compliances [21] and of posture on transpulmonary pressure [28], [29]. Thus, all methods can be used interchangeably when the focus is to detect changes of the parameters, rather than absolute values, among different ventilation conditions.

Finally, several practical limitations and advantages of each of the P_es_ measurement methods should be considered. While for air- or liquid-filled catheters only a correct positioning is necessary, for the balloon an appropriate choice and testing of the amount of air to be injected is required for a reliable measurement [5]. Also, while for the balloon measurements a specific catheter is required, a common nasogastric catheter might be used for liquid- or air-filled measurements [21]. Furthermore, while for balloon catheters the measurement is affected by several factors such as the amount of injected air, thickness, and dimensions of the balloon [30] − possibly resulting in differences between manufacturers and operators − for systems without balloon the only requirement to guarantee repeatability seems to be a sufficient rigidity of the catheter.

The major limitation encountered with air-filled catheter was the frequent worsening of the signal quality and possible occlusion of the catheter. Reversing this situation (flushing repeatedly the catheter using an air-filled syringe) was, in our experience, a cumbersome procedure in most cases. However, the results found suggest that this limitation did not affect significantly the estimation of the parameters of interest.

Liquid-filled catheters possess the appealing feature that measurements can be readily performed in most intensive care units and operating rooms with the monitors and pre-calibrated disposable transducers routinely used for invasive blood pressure monitoring [20]. The present technology of these transducers grants a very linear response to pressure changes, low noise, and flat frequency response in the range of interest (far superior than what achievable with air-filled or balloon catheters). Also, the built-in system of flushing and continuous flow of liquid (of approximately 3 mL/h) permits easy removal of air-bubbles in the measuring system. It also allows to successfully flush the catheter when signal quality worsen or occlusion is suspected, which occurred only once in two animals during the experiments. Nevertheless, the limitations of liquid-filled catheters, which are known since the 1950s [22], and possibly prevented the adoption of this technique in applications other than pediatric, should not be underestimated. Firstly, movement of the subject (e.g. shivering) can introduce non-negligible artifacts in the measurement. Secondly, large breaths might result in vertical displacement of the esophagus and consequently of the catheter, generating a water column of variable height between the transducer and the tip of the catheter, which affects the measurement of the respiratory swings of P_es_. Thirdly, placing the transducer at exactly the same height of the tip of the catheter is not trivial (since the latter is not visible), which likely results in a water-column between the two, and consequently a fixed offset in absolute P_es_ measurement. However, while this limitation is clearly relevant for standing or sitting subjects, in supine, prone, and lateral decubitus patients we speculate that the bias can be limited using anatomical landmarks and is likely to be small compared to the unknown offset between P_pl_ and P_es_.

## Conclusions

In anesthetized and mechanically ventilated pigs, air-filled and liquid-filled esophageal catheters without balloon can be used interchangeably with the traditional catheter with balloon to estimate P_es_ related parameters when the aim is to detect changes between different conditions, but possibly not when the absolute value of the estimated parameters is of paramount importance. Due to a better signal-to-noise ratio, and considering its practical advantages in terms of easier calibration and simpler acquisition setup, liquid-filled esophageal catheters without balloon may prove interesting for clinical use.
